# Risk Perception and Protective Behavior in the Context of COVID-19: a Qualitative Exploration

**DOI:** 10.1007/s41649-021-00181-3

**Published:** 2021-08-04

**Authors:** Salma Siddiqui, Azher Hameed Qamar

**Affiliations:** 1 Department of Behavioural Sciences, School of Social Sciences and Humanities, National University of Sciences and Technology, Islamabad, Pakistan; 2 School of Social Work, Lund University, Lund, Sweden

**Keywords:** COVID-19, Protective behavior, Coping, Religious coping, Risk perception

## Abstract

As a result of the devastating health effects of the COVID-19 outbreak, the lockdown has been considered a safety measure in many countries. In Pakistan, the first case of COVID-19 was reported in February 2020. The purpose of this qualitative study was to investigate people’s risk perception and protective behavior during the lockdown. Twenty-two (22) participants from eight big cities across Pakistan were interviewed. A six-step reflective thematic analysis was used for data analysis. The study focused on risk perception and protective behaviors. Our main analytical goal was to understand how risk perception shapes human behavior in the context of lockdown, pandemic-related information flow, and corresponding meaning-making. The study revealed that people influenced by information and advice campaigns form a perception of risk that has shaped their protective behavior. They used familiar means of coping with distress, including the search for strength through religious belief practices and following the precautions recommended by health professionals through the media.

## Introduction

The rapid spread of COVID-19 and the damage it inflicts on the physical and mental health of individuals are feared. The forced quarantine and lockdown to combat the disease have restricted social mobility and economic activities. Particularly in developing countries facing economic challenges, the complexity of human life has increased on a physical and psycho-social level. A literature review on the psycho-social impact of COVID-19 reported the alarming impact of the disease on human behavior, mental health, and social lives (Dubey et al. 2020). Besides, the economic crisis is also well documented, providing evidence of the negative effects of COVID-19 on human growth and development. A significant and direct relationship between human well-being, human growth, and development reflects how the COVID-19 has increased human suffering in the developing world (Feyisa 2020).

COVID-19 is a recently discovered disease caused by a novel coronavirus from the family of Severe Acute Respiratory Syndrome (SARS) (WHO 2020). It was first reported in Wuhan, China, and subsequently spread worldwide through human-to-human transmission. WHO declared COVID-19 a Public Health Emergency of International Concerns (PHEIC) on 30 January 2020. Protective measures recommended by WHO (2020) (i.e., Physical. Physical/social distance, disinfecting body and surfaces, and using masks) help combat the COVID-19 disease. Fear, an instinctive human emotion, helps in regulating behavior and coping with the situation effectively (Steimer 2002). For example, fear of COVID-19 promotes protective behaviors such as frequent hand washing and wearing a mask. However, extreme fear of COVID-19 may have damaging consequences for the individuals (such as social anxiety and phobias) and the society (such as xenophobia and panic buying). On the social level, the lockdown was seen as a safety measure in several countries. Even though it prevented the spread of disease, the prolonged lockdown caused detrimental effects on the economy and the psycho-social well-being of the people (Asmundson and Taylor 2020; Mertens et al. 2020; Zheng et al. 2020). The absence of a cure and the rapid spread of COVID-19 have altered the global picture, resulting in a sharp decline in socioeconomic activities and a critical need for healthcare facilities (to ensure protection and treatment). As a result, the WHO Scientific and Technical Advisory Group for Infectious Hazards (STAG-IH) recommended close monitoring and intensive surveillance, as well as an enhanced and rapid information system, to provide awareness, containment, and resilience improvement (Gatera and Pavarini 2020).

Pakistan shares its border with India, Iran (with a high death toll after Italy), and China (the epicenter of the COVID-19 outbreak). On 26 February 2020, Pakistan’s Ministry of Health confirmed the first COVID-19 case. On 6 April 2020, the total number of confirmed cases in Pakistan was 3277. The mortality rate was comparatively low (1.3%) and the recovery rate was high (4.8%). In 2021, by 12 June, 458,992 confirmed cases were reported. The cases were to rise in April 2021 and gradually decreased in June 2021 (on 23 April 2021, 5908 cases were reported, whereas on 12 June 2021, the reported cases decreased to 1239).

Pakistani government worked on National Action Plan for Preparedness and Response to Corona Virus Disease to develop the community emergency plan for an effective response to impending COVID-19 effects on public health. Several intensive media campaigns were launched to educate people about the symptoms of the disease and preventive measures. However, violation of corona standard operating procedures (SOPs) and limited capacity of the healthcare system is a challenge to overcome the healthcare crisis in Pakistan (Khalid and Ali 2020).

The current study explores people’s risk perception and protective behavior. Through qualitative investigation, this research will provide foundation for further qualitative and quantitative research in Pakistani and other similar sociocultural contexts. In this study, the following research questions were investigated.

1. How do people perceive risk related to COVID-19 breakout?
2. How do they protect themselves and others from COVID-19?

## Method

Qualitative research, as a descriptive and interpretative approach to investigate humans in their social context, is recognized as an important tool to understand hidden meanings and the depth of the empirical data by interpreting the process, experiences, events, and perceptions (Willig 2001). We used a descriptive qualitative research design with an inductive inquiry approach in this study. Semi-structured interviews were conducted to collect data and Braun and Clarke’s thematic analysis approach was employed.

### Context and Timing

The COVID-19 outbreak has devastating health impact and several countries viewed the lockdown as a safety measure. In Pakistan, the first COVID-19 case was reported in February 2020 and subsequent cases began to be reported across the country. The number of cases increased in March 2020, and few deaths were also reported. Therefore, on 23 March 2020, provincial-level lockdown for 15 days was imposed in various areas of Pakistan. Marriage halls, educational institutions, parks, playgrounds, gyms, malls, businesses, and markets were closed. However, the situation was not good. The number of confirmed COVID-19 cases on 30 April was 15,759 with 346 deaths; whereas 874 new cases were reported on 30 April (compared to 178 on 1 April). Hence, the lockdown was extended until 9 May 2020. On 9 May, a lockdown on fewer businesses, markets, and labor work was lifted to help lower-income people to survive in the current pandemic. The infection rate raised markedly after the lockdown lifted. Therefore, the government has decided to enforce a smart “lockdown” on 13 June 2020, in the identified hotspots. A “hotspot” was described as an area of elevated disease incidence or transmission, generally more than 1.5 cases per 1000 (Ghaffar et al. 2020). The residents living in these areas were asked to stay at home unless they need to visit a doctor or to buy groceries. The second wave of COVID-19 started at the end of October 2020, and the government continued its smart lockdown strategy and open business, marriage halls, parks and allow gathering by keeping social distancing and following other SOPs. However, the third wave that began in March 2021 has forced the government again to impose lockdown and close markets, education institutes, and businesses all over Pakistan.

### Sampling and Data Collection

Purposive sampling was used to access 12 women and 10 men (age range = 25––44 years, M = 32) living in eight big cities of Pakistan (Table 1). All the participants in the study belong to middle-class families (households). Middle-class households, as referred to in this study, are the households with an average monthly income of 50,000 PKR, and who can afford the basic facilities (water, electricity, gas, housing). It was also ensured that participants should have completed at least high school. None of the participants reported any chronic illness (such as asthma, hypertension, diabetes).

**Table 1:** Group data of study participants

Characteristics		Number
Gender	Females	12
	Males	10
Age	25–34	17
	35–44	5
Education	Graduate	9
	Undergraduate	12
	High school	1
Job status	Working	16
	Non-working	1
	Housewife	5
Cities (Pakistan)	Islamabad	2
	Lahore	4
	Karachi	4
	Peshawar	4
	Quetta	4
	Bahawalpur	3
	Multan	1

We planned to collect data from all the major cities in Pakistan. Our colleagues and former students (who lived in those cities) made contact with people who were willing to participate in the study. We obtained verbal consent over the phone by informing them about the study and their rights to participate voluntarily or withdraw anytime during the process. Interviews were scheduled based on the availability of the participants. To ensure the safety of the participants and researchers, all the interviews were conducted online using Skype, Zoom, and WhatsApp (according to the participants’ ease of access and use). All the interviews were conducted in April 2020 when the pandemic was new, and cases were on the rise. Hence, the interviews provided the first voices of the participants when they encountered the pandemic for the first time.

A semi-structured interview guide was developed based on relevant literature, the current situation in Pakistan as reported in print and electronic media, and researchers’ reflections on the current situation and potential participants. The interview guide consisted of ten questions inquiring about the risk perception of the current situation and corresponding protective behavior. All the interviews were conducted online and in the Urdu language (the national language of Pakistan, understood and spoken all over the country).

### Theoretical Stance and Process of Analysis

Employing Houston’s (2001) interpretation of critical realism, we understand that human subjectivity is shaped by the social structures, simultaneously recognizing individuals’ meaning-making through interaction with and within the context. In the context of the COVID-19 pandemic, the lockdown measures, campaigns, policies, enforcement, and reinforcements are shaping human risk perception and protective behavior. Remaining healthy (in the uncertainty and ambiguity of the current situation) is not a single phenomenon in the social world. Several mechanisms are operating at different levels of human experience ranging from the structure of the natural world to human resilience and social conditions where they seek health through protective behavior. Hence, an inductive qualitative analysis can unveil the interconnection between underlying psycho-social mechanisms and structure shaping human perception and behavior in the context of the COVID-19 pandemic.

Positioning us as researchers (one from mental health research and another from social work research), and our first-hand experience of lockdown in Pakistan, we were able to bracket assumptions, acknowledge disciplinary understanding, and recognize ground-up investigation in a data-centered approach to interpret the findings.

A back-and-forth examination of the data set, coded extracts of the data, and inductive analysis are all part of the thematic analysis. A six-step reflexive thematic analysis (Braun and Clarke 2006) was used to conduct this investigation. We used the checklist (adapted from Braun and Clarke 2006) to examine and strengthen the quality of data analysis in order to improve the analytical rigor of the analysis (Table 2). The transcripts were analyzed by both researchers in order to identify and discuss themes, common underlying structures, psychological mechanisms, and emerging patterns. To achieve dependability and conformability, the researchers ensured the procedural rigor, logical flow, and consistency of the interpretation. A detailed description based on the data’s semantic and latent meanings ensured transferability (case-specific generalizability).

**Table 2:** Thematic analysis check list

1	Transcription	Data has been transcribed in detail—a cross-check with the recorded data
2	Coding	Coding has been done taking all data into equal consideration—codes are driven by the data
3	Themes	Themes are generated from the codes and are comprehensively grounded in the data. They cross-checked against each other and reverse check with the original data. Themes represent an internal coherence as well as distinctiveness
4	Significant statements	Themes—relevant significant statements are sorted carefully and correspond to the themes
5	Analysis	The analysis is more than a description. It involves the sense-making of the data and provides a conceptual depth of the themes. Analysis connects the research questions with the themes and significant statements and provides a well-organized evidence-based analytical story
6	Reporting	Research has been reported adequately. It shows consistency among the research objectives, questions, method, and analysis. Researchers are active in the process and reporting (i.e., themes are emerged and interpreted with reflexive journaling)

## Results

Our reflexive thematic analysis revealed six overarching themes representing risk perception and protective behavior (Table 3). The results presented here define and interpret themes in the context of critical realism.

**Table 3:** Categories corresponding research questions

	Themes	Sub-themes
1	Risk perception	1. Fear of contagion and human vulnerability 2. Situating “others” irresponsible behavior
2	Protective behavior	1. Personal hygiene 2. Physical distancing 3. Religious coping

### Risk Perception

Risk perception is a subjective psychological construct depending upon psycho-social factors varying across individuals and groups. Even though the cognitive evaluation involved in risk perception depends on information sources, exposure to the risk, and people’s intention to believe and follow the information, the emotional response engage our risk perception with our fears (Leiserowitz 2006; Sjoberg 2002; van der Linden 2017). Risk perception also depends on the trust in the authority providing information about the risk, familiarity with the situation, awareness about the risk, and perceived uncertainty. In the context of the COVID-19 breakout, the critical situation around the globe requires studying the risk perception of the people regarding this infectious disease. People’s perceptions of risk associated with the fear of contagious coronavirus may inform us about their protective measures and coping strategies (Van Bavel et al. 2020). For example, one of the participants in our study talked about her initial information about the COVID-19 breakout and its symptoms that she later reflected in her protective behavior.

This is a pandemic. I understand how painful this is. First, I would say, if this pandemic had only affected the developing countries like ours; I might have interpreted it as the lack of healthcare facilities. However, this is worldwide and even superpowers are powerless to stop it. It means it is difficult to control and we cannot ignore this. We are being told by the media that we should reduce our social interactions, and we should almost completely disconnect ourselves from the outside world. We have no idea. We do not know who is safe or not. We must stay at home. We can disconnect ourselves from the outer world. Second, they are informing us about the symptoms such as sore throat, flu, breathing difficulties, and fever. People above fifty years of age, children below five years of age, and people with low immunity are vulnerable.

The participant shared the information she obtained from the media about the symptoms of the disease and the vulnerable population. Developing countries generally lack resources to meet the healthcare needs compounded by over-population, under-developed public health infrastructure, and poor or limited capacity to handle medical emergencies. Informed by the media, the participant views the global pandemic as “uncontrolled” which developing countries were not able to manage despite their resources and infrastructure. The way she inferred the “uncontrollability” of the disease in the context of global effects of the pandemic, she reflected her belief in the information provided to her through various sources that shaped her risk perception and corresponding precautionary measures. Ease of access to media and the magnitude of timely information provided by the popular media have an impact on general risk perception at both the personal and societal levels. Personal risk perceptions may be influenced by cultural and traditional sources which may limit behavioral change caused by media-based general risk perception (Oh et al. 2015; Vai et al. 2020). However, the participants in this study (who are new to the current situation) are looking for information to help them to protect themselves. The media campaigns supported by government and internationally recognized healthcare institutes and agencies have an authoritative influence directing a cognitive dimension of the risk associated with COVID-19 breakout.

Participants expressed their concerns about the contagious disease, human vulnerability, and government response in the context of the COVID-19 breakout and prevailing lockdown situation. At the same time, participants situate other people as “irresponsible” while discussing about their own protective behavior.

#### Fear of Contagion and Human Vulnerability

The participants’ understanding of coronavirus was influenced by their consumption of the “popular” newsfeed via electronic and social media. They shared their awareness of the highly contagious properties of the coronavirus and expressed concerns about its easy and rapid transmission through the air and physical contact.

The virus is extremely contagious. It spreads quickly by shaking hands with the infected person or touching objects. If it is in the air, it can enter into your body through the nose. Now I have learned (from the media) that this virus could live in your wallet or mobile phone. That is, in addition to sanitizing hands, we must sanitize keys, wallet, cell phones, etc. (p14)

With the growth of the disease outbreak, people became interested in the disease and learned about its infectious effects in the media.

Now we know that the coronavirus is spreading and that hospitals are running short of treatment facilities, we are eager to understand this phenomenon. Whatever we have learned from the media and medical professionals about its highly contagious properties is now a public knowledge. It has the potential to contaminate the air we breathe; this is alarming. (p7)

Despite the fact that the participants in this study had no direct experience or direct observation of COVID-19 cases, they appear to accept the “truth” as documented in the news media and country status reports. Therefore, as findings reflect, information and information sources are structured to learn the fear of contagion and the associated human vulnerability. Based on the news related to the uncertainty of the disease and the non-availability of any cure or treatment, participants perceived coronavirus as a real threat to human health. It was also discovered that at first some participants did not consider the disease to be anything more than the flu caused by a cold or allergic reaction (such as dust, pollution). However, later with the gradual increase in the cases (as disclosed by the government) and with a rise in sensitization of the issue by the media, the risk perception associated with high transmissibility and incurability of COVID-19 also increased. For example, one of the participants talked about his earlier misconception about the disease.

We initially assumed that this was just another bad case of flu that turned into a sore throat and could be treated with standard home care and medications. According to reports, its symptoms were similar to contagious allergic reactions, such as sneezing and running nose. When more cases were reported, and we learned that severely ill patients had been admitted to ICUs (intensive care units), we realized this was not a typical flu.

According to the findings, participants used their perceptions of “responsible” behavior to situate the “irresponsible” behavior of others. As a result, participants appeared to use their risk perception as a standard to judge the “irresponsible” behavior of others, which also triggers their sense of risk and fear.

#### Situating “Others” Irresponsible Behavior

As previously stated, participants viewed other people’s behavior as “irresponsible” because they do not see them understanding the risk of disease exposure and being aware of its highly contagious effects. They viewed other people’s actions through the lens of risk perception. Other people’s “irresponsible” behavior was also perceived as a challenge. People who do not take precautionary measures (such as keeping a safe distance and wearing masks) make it difficult to control or limit the pandemic; for example, participants expressed concern about the careless response of those around them.

On the one hand, the government is attempting to educate people about the gravity of the situation and advising them on all possible precautions; on the other hand, most people are ignoring these precautions. They are completely unaware of the dangerous situation. As a result, they lack a sense of responsibility in this dire situation. They are irresponsible in their behavior and cause problems for many others.

They emphasized the importance of altering their daily routines in terms of physical distance and the use of masks.

People, for example, are not willing to change their routines, which means they continue to spend their social lives in the same way. They go to markets on a regular basis. They are leaving the house unnecessarily. Wearing a mask is not common, but it should be. Physical distance is not yet accepted as a precautionary measure, and people do not avoid shaking hands and getting too close in the streets and markets. Overall, they are unconcerned about the situation and are treating it casually or as usual. (p12)

The participants identified people’s behavior as “irresponsible” and “pathetic” in the realization of the grave situation caused by their carelessness.

The public behavior is pathetic, and they are not ready to take it seriously. They do not have any idea about its severity. A few days ago, in an online video, a medical doctor was appealing to the public to stay at home. Our people are not serious about it. Some educated people are conscious of it and taking measures. However, several educated people make a crowd at grocery shops. This is pathetic. (p13)

Another reason participants mentioned the irresponsible behavior was the poor understanding of the symptoms of the disease as people mistook it as the common flu or cough. Hence, people who are used to follow folk remedies do not follow government instructions, such as repeated handwashing, use of masks, sanitizers.

We should not use any home remedies. We must take precautions such as washing our hands and maintaining our cleanliness. People do not take the disease seriously and take things for granted. They are unaware that this disease is not the same as the flu and cough that they used to treat without taking precautions. (p16)

Other reported concerns about irresponsible behavior included denial of the situation, intentional negligence, a lack of awareness, being non-serious, and being uncooperative. Participants expressed their concerns about the rapid spread of COVID-19 while discussing their protective behavior and contrasting it with that of others who were “irresponsible.” The way all participants perceive other people’s “irresponsible behavior” in comparison to their own sense of responsibility (which they gradually learn) also demonstrates that the participants who agreed to participate in this study are those who believe COVID-19 is a reality.

### Protective Behavior

As previously stated, the structure of the top-down flow of information influences the construction of meanings of the risk associated with COVID-19, and participants’ risk perception in this context constructs the meaning of “irresponsibility.” Similarly, we identified three themes related to protective behavior reflected in the underlying structure of lockdown, awareness campaigns, and government and private media sensitization of the situation. Our research uncovered three types of protective behaviors. The physical level measures (personal hygiene); the social level measures (physical distancing as directed by the government and healthcare professionals); and the third level measures (religious coping, which is a cognitive reappraisal of the stressful event, human limitations, and corresponding religious beliefs). Participants reported their coping strategies used during the lockdown under the umbrella of “responsible behavior.” Participants discussed their precautionary measures to demonstrate their responsible behavior in the face of the ongoing pandemic. That is how they use their self-reported responsible behavior as a standard to judge “other people’s irresponsible behavior.” For example, one of the participants said:

People who are aware of the severity of the pandemic are taking reasonable precautions to protect themselves and their families. They are following information from various media sources and will continue to do so. (p13)

It was clear that participants understood “sensible behavior” as following instructions to seek protection.

I’m following the instructions given to me by the media. My family and I have been quarantined at home. I’m not going outside unless absolutely necessary. When I go grocery shopping, I put all of the bags in a corner of the room for 24 hours. In the case of perishable goods, I thoroughly wash them. I don’t let my kids touch anything until I’ve properly disposed of it. I wash my hands and put on new clothes. To boost my children’s immunity, I give them vitamin supplements and fruits. (p1)

Aside from religious coping, which we will discuss later, the findings revealed personal hygiene and physical distancing as protective strategies that participants use in accordance with healthcare professionals’ and government media campaigns’ instructions.

#### Personal Hygiene

We are sanitizing everything that has physical contact with the person coming from outside. We are, for example, sanitizing doorknobs, switches, chairs, and tables. Whoever goes out has a separate set of clothes and shoes that he or she must change after returning home. We are especially concerned about children, and they are frequently asked to wash their hands in this situation. Children, you know, carelessly touch everything. On the other hand, we avoid ready-made foods and limit ourselves to dining out and takeaway food. In this case, home cooking is the best option. All we can do is try to protect ourselves. We recognize that this is not perfect, but given the circumstances, we can do our best. (p17)

Participants reported personal hygiene as an early and important protective measure against COVID-19. Whatever they learned from healthcare campaigns in the context of COVID-19, they practiced it in several different ways, such as disinfecting the body, clothes, vegetables, and fruits with water and chemicals. The primary objective is to use personal hygiene as a scientific shield against coronavirus.

I am very careful now. For example, I went to a bakery, and I did not touch the door handle. I pushed the door open with my elbow. My son was also with me. After coming home, we washed our hands with soap. Then I managed the stuff we bought. Washing hands with soap is compulsory nowadays. I do not let my kids to touch anything without washing hands with soap, especially after coming home. (p16)

One participant talked about how she is trying to manage hygiene behavior at home while instructing children and her husband.

Whenever my husband comes home, I ask him to take a shower to disinfect him. I wash his clothes with disinfectant chemicals and detergent, and dry them in the sunlight. Children need more specific instructions. I pay attention to their activities at home and outside. They are strictly advised to follow the instructions. (p13)

Participants described their hygiene practices as learned routine actions related to their COVID-19 knowledge and concerns. It was clear that they attempted to modify their behavior in accordance with the instructions they received through the media. The emphasis on personal hygiene appeared to play a critical role in reducing their anxiety. We discovered increased use of antiseptic and disinfectant chemicals, frequent handwashing, and deliberate efforts to adapt hygiene behavior.

#### Physical Distancing

WHO recommends physical distance as a preventive measure. Physical distancing entails keeping at least six feet away from other people in public places (such as markets, hospitals, public transport). Participants limited their social interaction in order to maintain physical distance. They avoided social and religious gatherings, as well as unnecessary outings.

We are physically cut off from everyone. We use social media to communicate with our friends and family. We’re avoiding social gatherings. At home, we’ve also stopped having our maid come in and clean the house. We are avoiding unnecessary trips to markets and stores. My husband buys groceries for the entire week, making sure to have as little physical contact with people as possible. If someone pays us a visit, we make every effort to avoid handshakes. In any case, there are some things we simply cannot avoid out of respect, such as shaking hands with the elderly. (p2)

Participants attempt to maintain physical distance; however, in their cultural context, this is interpreted as social distance. As previously stated, refusing to shake hands with elderly people may be perceived as disrespectful. Similarly, people offering Salat (the five-times-a-day prayer ritual practiced by Muslims) at mosques should pray in rows (as required to offer the ritual prayer). In this situation, it is difficult to avoid close contact. Male Muslims are strongly encouraged to pray at the mosque, while female Muslims are not required to do so. One of the male participants used the COVID-19 precautions to justify praying at home and avoiding physical contact with other people in the mosque.

Yes, some people believe we should go to the mosque, and the mosque should not be empty during prayer time. People, however, cannot maintain physical distance while praying together. To offer prayer, all participants must stand in a row. In this terrible situation, praying at home should be sufficient. Even the holy cities of Makkah and Madinah in Saudi Arabia are closed. It teaches us that we can pray at home without having to go to religious gatherings at the mosque. (p14)

The participants practiced physical distancing while compromising on several culturally expected social interactions. On the other hand, avoiding unnecessary market or public place visits, as well as limiting receiving services at home (such as maids), was a preventive measure to ensure limited physical contact with people. One participant mentioned three protective measures that she was using and thought they were useful.

We are taking the most stringent precautions. First, wash hands frequently; second, avoid handshakes; and third, stay at home the majority of the time. We are trying to do so in order to avoid the pandemic. We avoid gatherings for meals, parties, and religious reasons. As recommended by the healthcare professionals, yes, it is helpful. At least, we know we are protecting ourselves and others by taking precautions. (p15)

The steps they take to protect themselves against the coronavirus (as advised by media campaigns) provide them with a sense of security, which ultimately helps to reduce anxiety in this critical situation.

#### Religious Coping

It is my belief and I think every Muslim believes in it that this is a natural disaster. Allah is showing us what he can do. He is informing us about our limitations and vulnerability in this world. And in our prophet’s lifetime when a pandemic struck a region, the prophet (peace be upon him) advised people not to leave the infected area and do not enter the infected area. It implies that we should be careful. But it is also true that death is the ultimate destination no matter wherever we are. Hence, we must seek Allah’s forgiveness. We should pray and seek his shelter. This (pandemic) is from him and he will rescue us. (p16)

The majority of the participants discussed their religious coping strategies for seeking divine protection during the COVID-19 pandemic. Participants reported common religious practices such as connecting to their faith, turning to religious teachings to maintain health, ritualized cleaning, and prayers. They discussed connecting with their faith in God and seeking his protection. This faith-based shield, they say, gives them hope that they will be safe during the pandemic. One of the participants used the story of the prophet Jonah, who was swallowed by a whale as an example.

We must pray to Allah. Recite His praises and glory, and beg forgiveness from Him. ‘Ayat Karima’ must be recited (the verse in the Quran that Prophet Jonah recited when he was in the belly of the whale). It was due to the miracle of Ayat Karima that prophet Younus (Jonah) survived in the belly of the whale until it spit him out at the shore. If we ask Allah for forgiveness. I believe that repeatedly reciting *Ayat Karima* will eventually rescue us from this terrible situation. (p16)

*Ayat Karima* is a verse in Quran that says, “There is no deity except You; exalted are You. Indeed, I have been of the wrongdoers.” Participants mentioned several verses from the Holy Quran that they believe to work as a protective shield against unseen hazards, threats, and diseases. All these verses are about praising God, submitting to his will, accepting human vulnerability, seeking is forgiveness and shelter.

The findings also revealed that participants get religious reinforcement to practice personal hygiene. For example, doing ablution to offer prayer five times a day is perceived as a purification ritual necessary to offer prayer ritual.

We believe that only Allah can protect us. Our religion directs what we should do. We need to keep ourselves clean. We should perform ablution to cleanse ourselves physically and spiritually. We can’t offer prayer unless we’ve had ablution. Hands, nose, ears, face, arms, and feet should be thoroughly washed five times a day to maintain personal hygiene. Then, praying is a way to connect with God and ask for his blessing. (p11)

Participants expressed faith in God as the ultimate power. During the current pandemic, they expressed feelings of helplessness and submission in order to seek protection. Seeking satisfaction through religious rituals was a faith-based behavior that participants perceived as beneficial in dealing with stressful situations. In some ways, the pandemic has helped religious people reconnect with their faith with renewed vigor. In some cases, it is a declaration of their faith as the most valuable and beneficial resource in their lives. Overall, our findings revealed religious practices to be an effective coping strategy for remaining resilient during this pandemic.

## Discussion

As described in the beginning, the Pakistani government did not impose a complete lockdown in the country. Considering the economic challenges that may bring more disasters than COVID-19, the Pakistani government preferred smart lockdown imposed in worst-hit areas. However, there has been an intensive media campaign (print, electronic, social media) to build an awareness environment that ultimately got a positive response with the exposure of the severity of the issue. Although it was not intended, participants in this study were all those who were following the instructions given by health experts and information broadcasted through the media. Their perception about the risk associated with coronavirus was linked simultaneously with their sense of vulnerability and structural information they were following every day.

**Fig. 1:**
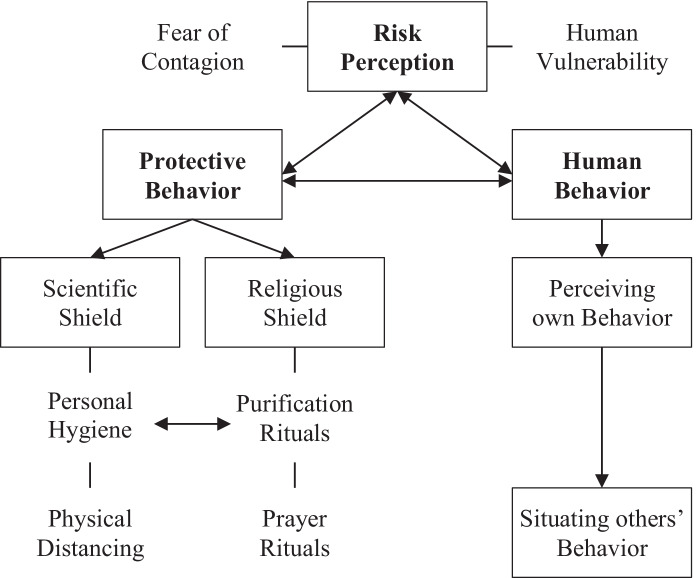
Risk perception and protective behavior—thematic map

As illustrated in Fig. 1, risk perception includes the sense of human vulnerability and a fear of contagion. Our analysis revealed the interconnection between human behavior and risk perception. Participants’ risk perception was a combination of their understanding of the human body (that they disclosed as vulnerable and exposed to external threats, such as germs) and their “trust” in the information they were receiving which induced the fear of contagion. It is also true that participants were already aware of contagious diseases such as flu. However, in the case of COVID-19, they construct their meanings of contagion in connection with their exposure to information and their gradually improved reception to information.

Risk exposure, sensitivity, and adaptive capacities are interrelated aspects of human vulnerability (Adger 2003), and several internal (e.g., socioeconomic conditions), external (e.g., natural disasters), and structural (e.g., infrastructure) stress factors affect human vulnerability (Menezes et al. 2018). The findings of the current study provide a conceptualization of vulnerability (in connection with COVID-19 in Pakistan) in the context of risk perception that corresponds to the exposure to risk on a structural level and human behavior at the community level. Risk perception may lead to personal decision-making depending upon the individual’s frame of reference; however, it is not independent of emotional, social, cultural, and cognitive factors (Leiserowitz 2006; Joffe 2003). Our analysis of perceived risk associated with COVID-19 reveals a triangular relationship between risk perception, human behavior, and protective behavior (Fig. 1). Participants discussed their “responsible” behavior while they perceive “responsibility” as the intention and ability to follow the healthcare instructions. Furthermore, they used their behavior and practices as a standard to situate others as “irresponsible.” Hence, adherence to preventive protocol shaped the notion of “responsible behavior” and risk perception during the pandemic.

Risk perception and corresponding responsible behavior are closely linked to protective behavior and coping mechanisms. The two dimensions of protective behavior (physical measures and religious coping—as a cultural component) interconnect to provide a self-administered coping mechanism that assisted participants to go through apparently uncertain, yet transitory experiences of fear of COVID-19. This coping mechanism exists at the intersection of the structural aspects of COVID-19 (as seen in lockdown and instructional campaigns) and the meaning-making of the participants using COVID-19 as a frame of reference. All protective behaviors (with the exception of religious coping) are based on the awareness generated through the media and public/private healthcare campaigns corresponding to the spread of COVID-19. These campaigns educate people about the risk of ignoring the precautions and the advantage of consistent adherence to protective measures based on scientific evidence. Risk-oriented consistency later shaped some of the behaviors that had a positive impact on their lives, such as personal hygiene. The understanding of the specific health hazard and human vulnerability triggered the protective behavior. In critical situations, individuals perceive vulnerability through the sources of information that can create a positive or negative cognitive appraisal of the situation and the adoption of various coping modes to seek protection (Clubb and Hinkle 2015; Khosravi 2020). Hand washing, mask wear (when going out), and handling and disinfecting grocery items were all reported as routine practices and responsible behavior during the lockdown. It was clear that a general understanding of infectious diseases (such as flu) had gradually become sensitized as a result of media coverage and the spread of COVID-19 in the participants’ surrounding areas. The participants perceived these measures to be “rational,” based on scientific explanations of the disease and its spread. Though the overall physical measures were seen as more practical, providing a scientific shield against COVID-19, a cognitive “restlessness” was present, caused by the increasingly uncertain and stressed situation duly addressed in return by the positive cognitive reappraisal of religious beliefs and adopting religious coping (conceptualized here as a cultural shield).

Religious coping appears to be a reaction to the situation’s uncertainty and anxiety caused by unexplained or ambiguous threats. Several studies on patients have found religious coping to be a unique component in stress management and acquiring the necessary physical and psychological resilience against the disease to survive stressful events (Koenig et al. 2001; Tix and Frazier 1998). The proclivity for religious coping is rooted in long-held cultural beliefs that come into play when people recognize their individual and collective limitations (Pargament and Raiya 2007). For example, one participant expressed feelings of helplessness, “I believe this is a warning sign from him. Let’s just say…we were busy in our life, and suddenly our lives have stopped. So… it is a sign from him that he has ultimate control, and we cannot.”

Helplessness and fear appear to activate the “religious” recognition of God, resulting in a religious feeling. Religious feelings appear to be associated with submission to a divine power and a desire for rescue. For example, submitting to Allah and seeking his forgiveness was a religious way to obtain divine assistance. The majority of participants described God as merciful, and religious beliefs and practices as coping strategies. They shared their connection with God as a beneficent being, which was reflected in their responses in which they associated their hygiene practices with religious teaching. We, therefore, see that religious belief provides a structure to reinforce people to practice personal hygiene, reported by the participants as “cleanliness is half of the faith.” They also “rationalized” the scientifically recommended hygiene in their routine purification rituals to offer prayer.

Throughout the Islamic world, the concept of powerful Quranic words is regarded as a fundamental religious impression (MacPhee 2003; Qamar 2016). Reciting the Quran (which Muslims believe to be God’s book) and offering Salat were seen as spiritual ways to gain psychological power to cope with anxiety. According to the findings of this study, religious expressions and sentiments instill fear (“death”) and hope (“life”) in the hands of the divine. While experiencing uncertainty in a health-hazard situation, the human-divine relationship was attributed as a shield for protection. As the religious experience emerges from the core religious beliefs, the situation’s uncertainty is more likely to be managed through the “inevitability” of the religious shield, alleviating fears and concerns. In this regard, the religious component contributes to a better understanding of the critical links between religious beliefs, risk perception, anxiety, and coping, all of which contribute to religious satisfaction and compliance (Fabricatore et al. 2000; Kaczorowski 1989).

We see the religious impact on people’s lives during this pandemic between the emergence of personal hygiene and purification rituals. An internal state of religiosity that typically arises during times of anxiety and helplessness provides faith-based strength to cope with the situation and future concerns. As previously stated, religious coping can be viewed in this study as a cultural shield that connects a person with his or her spiritual self, cultural group, and current scientific measures (especially personal hygiene). Our participants were all Muslims living in a country where Muslims make up nearly 97 percent of the population. Their spiritual selves were connected with shared core beliefs about divine existence, power, and control. The use of religious beliefs and practices, therefore, ritualized the cultural shield and religious provocation in dealing with anxiety.

## Conclusion

The study provides an exploration of risk perception and protective behavior among Pakistani people (belonging to middle-class socioeconomic status) in the context of COVID-19. At the earlier stage of the COVID-19 spread in the country (April 2020), awareness of risk and threat was influenced and regulated through print and electronic media. People learned from the news about the nature of the disease, and the government’s plans to contain the spread. With the gradual increase in COVID-19 cases and observable non-adherence to preventive guidelines, the participants expressed their fears and concerns situating the risk of exposure and other’s irresponsible behavior. As people’s knowledge about the COVID-19 is framed in the scientific paradigm of contagious diseases, the protective measures were also emerging from scientific evidence. Hence, the scientific shield was shaped by the structural interpretation of the participant’s knowledge about the disease which appeared closely adhered to the increased frequency of washing hands with soap, disinfecting surfaces and clothes, and medical compliance. The religious experience emerged as a cultural shield used to overcome the anxiety and stress caused by the uncertainty that was also informed by the media along with precautionary measures. The risk perception and coping behavior (Fig. 1) provides a qualitative exploration of human behavior in connection with risk perception and coping strategies. The derived thematic map provides a model to explore and interpret conceptual interconnections between risk perception and human behavior in the context of COVID-19.

## Implications of the Study

This study provides useful information and analysis of people’s protective behavior during COVID-19 in the Pakistani cultural context. The data was obtained from different cities of the country where the COVID-19 cases were reported. The findings revealed risk perception, protective behavior, and coping strategies. Researchers, practitioners, and other professionals interested in behavior study and behavior change during a pandemic can find this study as a useful source based on empirical data.

## Data Availability

The authors opt not to share data.
